# Aflatoxin B1: Challenges and Strategies for the Intestinal Microbiota and Intestinal Health of Monogastric Animals

**DOI:** 10.3390/toxins17010043

**Published:** 2025-01-17

**Authors:** Hyunjun Choi, Yesid Garavito-Duarte, Alexa R. Gormley, Sung Woo Kim

**Affiliations:** Department of Animal Science, North Carolina State University, Raleigh, NC 27695, USA; hchoi25@ncsu.edu (H.C.); yrgaravi@ncsu.edu (Y.G.-D.); agormle@ncsu.edu (A.R.G.)

**Keywords:** aflatoxin, chickens, growth performance, intestinal health, pigs

## Abstract

The objective of this review is to investigate the impacts of aflatoxins, particularly aflatoxin B1 (AFB_1_), on intestinal microbiota, intestinal health, and growth performance in monogastric animals, primarily chickens and pigs, as well as dietary interventions to mitigate these effects. Aflatoxin B1 contamination in feeds disrupts intestinal microbiota, induces immune responses and oxidative damage, increases antioxidant activity, and impairs jejunal cell viability, barrier function, and morphology in the small intestine. These changes compromise nutrient digestion and reduce growth performance in animals. The negative impact of AFB_1_ on the % change in average daily gain (ΔADG) of chickens and pigs was estimated based on meta-analysis: ΔADG (%)_chicken_ = −0.13 × AFB_1_ intake per body weight (ng/g·d) and ΔADG (%)_pig_ = −0.74 × AFB_1_ intake per body weight (µg/kg·d), indicating that increasing AFB_1_ contamination linearly reduces the growth of animals. To mitigate the harmful impacts of AFB_1_, various dietary strategies have been effective. Mycotoxin-detoxifying agents include mycotoxin-adsorbing agents, such as clay and yeast cell wall compounds, binding to AFB_1_ and mycotoxin-biotransforming agents, such as specific strains of *Bacillus subtilis* and mycotoxin-degrading enzyme, degrading AFB_1_ into non-toxic metabolites such as aflatoxin D1. Multiple mycotoxin-detoxifying agents are often combined and used together to improve the intestinal health and growth of chickens and pigs fed AFB_1_-contaminated feeds. In summary, AFB_1_ negatively impacts intestinal microbiota, induces immune responses and oxidative stress, disrupts intestinal morphology, and impairs nutrient digestion in the small intestine, leading to reduced growth performance. Supplementing multi-component mycotoxin-detoxifying agents in feeds could effectively adsorb and degrade AFB_1_ co-contaminated with other mycotoxins prior to its absorption in the small intestine, preventing its negative impacts on the intestinal health and growth performance of chickens and pigs.

## 1. Introduction

Mycotoxins are secondary metabolites produced by toxigenic fungi, and it is estimated that 25% of the world’s cereals are contaminated with mycotoxins [1,2,3]. Mycotoxins play crucial roles in a range of toxic mechanisms, compromising various metabolic functions in both humans and domestic animals [4]. Monogastric animals, including chickens and pigs, are particularly susceptible to mycotoxins, unlike ruminants that harbor a diverse microbiota capable of metabolizing and detoxifying certain mycotoxins in the rumen [5,6,7]. Chickens and pigs are of high interest not only due to their vulnerability to mycotoxins but also their significant roles in global meat and egg production [8,9]. As two of the most widely farmed livestock species worldwide, the negative impacts of mycotoxin contamination on the health and growth performance of chickens and pigs can have substantial economic and food security implications [9,10]. Among the diverse types of mycotoxins, aflatoxins have gained high regulatory attention and scientific focus due to their toxicity and prevalence [1,11].

The name “aflatoxin” is derived from “A” for *Aspergillus*, “fla” for “*flavus*”, and “toxin” for its toxic nature [12]. This indicates that aflatoxins are primarily produced by certain species of *Aspergillus* fungi, including *Aspergillus flavus* and *Aspergillus parasiticus.* There are four main types of aflatoxins, including aflatoxin B1 (AFB_1_), aflatoxin B2, aflatoxin G1, and aflatoxin G2. In particular, AFB_1_ is known as the most toxic mycotoxin to animals. A previous in vitro study reported that AFB_1_ has a stronger inhibitory effect on cell growth compared to other aflatoxins [13]. Additionally, mixtures of aflatoxins further enhance the inhibitory effects on cell growth [13]. Due to the severe health risks specifically associated with AFB_1_, AFB_1_ is highly regulated, especially in feeds that are more likely to cause aflatoxicosis, a toxic condition resulting from aflatoxin ingestion by animals [14]. In terms of prevalence, AFB_1_ is more commonly found than other aflatoxins in feedstuffs such as corn, corn co-products, peanut meal, and cottonseed meal [15]. These feedstuffs are often grown in warm and humid climates, which are conducive to fungal growth and aflatoxin B1 production, particularly during the pre-harvest and storage stages [15]. Lastly, *Aspergillus* fungi produce AFB_1_ more than other aflatoxins, indicating that the contents of AFB_1_ could be more pronounced in feeds [16]. As a result, AFB_1_ is the primary concern among aflatoxins in feed safety management.

The gastrointestinal tract (GIT) is the first biological system to come into direct contact with aflatoxins upon ingestion, and it is here that the negative impacts of aflatoxin exposure begin to manifest [17]. However, despite the GIT being the initial site of exposure, much of the research on aflatoxicosis has focused on systemic effects, such as liver damage and immune suppression, and the specific impacts on intestinal health have been less thoroughly explored. Understanding the impacts of AFB_1_ in the intestine is also crucial, particularly in the small intestine, as the small intestine is where mycotoxins would first affect the animal by altering intestinal microbiota and activating intestinal immune cells [18,19,20]. Additionally, AFB_1_, like nutrients, are primarily absorbed in the small intestine, making their effects particularly significant in this region [21].

Aflatoxin B1 has been shown to alter the intestinal microbiota, reducing beneficial microbial populations such as *Lactobacillus* spp., and simultaneously promoting the growth of harmful bacterial species such as *Escherichia coli* in the small intestine of animals [19,22]. The negative modulation of luminal and mucosa-associated microbiota in the small intestine also has a great influence on the intestinal health of animals [23]. These changes can further compromise intestinal health, weakening the immune defenses that are critical for protecting the animal from infections and diseases [24]. Aflatoxin B1 can also compromise intestinal integrity by decreasing the relative gene expression of tight junction proteins, which weakens these connections and leads to increased intestinal permeability [25,26]. This allows pathogens and toxins to enter the bloodstream more easily, resulting in systemic inflammation and immune challenges. Moreover, AFB_1_ can damage enterocytes, impairing the ability of the intestine to absorb nutrients effectively [26,27]. This not only leads to reduced growth performance but also exacerbates malnutrition in animals. This understanding is crucial for developing dietary interventions to mitigate the negative impacts of aflatoxins in feeds for monogastric animals. Therefore, the objective of this review is to investigate the impacts of AFB_1_ on the intestine and growth performance after absorption, and to comparatively evaluate various dietary interventions that could effectively prevent these negative impacts on intestinal health and growth performance in monogastric animals.

## 2. Influence of AFB_1_ on Intestinal Microbiota in Monogastric Animals

### 2.1. Intestinal Microbiota

The intestinal microbiota has gained increased recognition for its critical role in maintaining intestinal health and supporting growth in animals [28,29]. Beneficial bacteria in the intestine help prevent colonization by opportunistic pathogens or ammonia-producing bacteria, thereby reducing the risk of intestinal inflammation [30,31,32]. Aflatoxin B1 adversely affects the intestinal microbiota [33,34,35]. In pigs, AFB_1_ exposure has been associated with an increase in *Escherichia coli* populations within the colonic digesta [26] (Table 1). These adverse impacts on the intestinal microbiota are likely due to the reduced production of short-chain fatty acids, a consequence of diminished populations of fiber-degrading bacteria [36]. Additionally, AFB_1_ has been shown to promote pathogenic bacterial infections, including *Escherichia coli*, *Salmonella*, and *Klebsiella*, in the ileal digesta of chickens [22]. This rise in infections is likely compounded by immune suppression caused by AFB_1_, which makes the intestine more susceptible to pathogenic invasion. Both the luminal and mucosa-associated microbiota (also called ‘mucosal microbiota’) in the small intestine play a critical role in preserving intestinal barrier functions, thereby preventing translocation of harmful pathogens and toxins to enterocytes of animals [33,37]. The mucosa-associated microbiota, however, directly interact with the mucus layer and intestinal cells [38,39,40], indicating the changes in the mucosa-associated microbiota could provide substantial evidence of the negative impacts of AFB_1_ on epithelium cells in the small intestine of animals. The difference between the luminal and mucosa-associated microbiota is distinct [41]. Therefore, conclusions drawn from findings examining only the luminal microbiota could lead to misunderstandings about interactions between the intestinal microbiota and the intestine. Additionally, AFB_1_ increases the relative abundance of *Staphylococcus* and *Escherichia-Shigella* and decreases *Lactobacillus*, *Burkholderia caballeronia paraburkholderia*, *Romboutsia*, and *Corynebacterium* in the jejunal tissue of chickens [19]. Similarly, AFB_1_ increases the prevalence of *Staphylococcus xylosus* in the jejunal tissue of chickens [42]. In nursery pigs, AFB_1_ contamination in feeds also negatively affects the mucosal microbiota in the jejunum, reducing *Lactobacillus kitasatonis* [43]. Thus, changes in mucosa-associated microbiota by AFB_1_ are an important parameter to assess the influence of aflatoxins on the intestinal health of animals.

### 2.2. Crosstalk Between the Mucosa-Associated Microbiota and the Intestinal Immune System

Crosstalk between the mucosa-associated microbiota and the intestinal immune system is critical for maintaining mucosal homeostasis. The small intestine of animals contains receptors on immune cells, such as dendritic cells and microfold cells in the epithelium, which recognize intestinal antigens primarily through toll-like receptors (TLRs) and nucleotide-binding oligomerization domain-like receptors (NODs). These pattern recognition receptors modulate cytokine production to maintain the immune function and intestinal health of animals [23,44]. An increase in cytokine production triggered by AFB_1_ leads to heightened pro-inflammatory cytokine production and oxidative damage within the jejunum of animals [26,45]. A previous study reported that 40 µg/kg of AFB_1_ in feeds increased the relative abundance of pathogenic bacteria, such as *Staphylococcus*, whereas it decreased the relative abundance of beneficial bacteria, such as *Lactobacillus*, in the jejunal tissue [19]. Additionally, AFB_1_ increased the relative mRNA abundance of TLR2, NOD1, inducible nitric oxide synthase (iNOS), and nuclear factor kappa-light-chain-enhancer of activated B cells (NF-κB) as well as increased the relative mRNA abundance of interleukin-6 (IL-6), interleukin-8 (IL-8), and tumor necrosis factor-alpha (TNF-α) and increased cysteine-aspartic acid protease-3 (CASPASE-3) protein expression in the jejunum of chickens [19]. An in vitro study using 200 µg/kg of AFB_1_ similarly increased the relative mRNA abundance of NF-κB and iNOS, elevated pro-inflammatory cytokines including IL-6, IL-8, and TNF-α, and decreased intestinal epithelial cell viability in chicken embryo primary intestinal epithelium [20]. These findings suggest that AFB_1_ is strongly correlated with changes in the mucosa-associated microbiota and epithelial cell receptors, which can induce immune responses and affect the viability of small intestinal cells in animals. However, another study reported that 600 µg/kg of AFB_1_ reduced the mRNA expression of TLR2-2, TLR4, and TLR7 in the jejunum [46], indicating that excessively high AFB_1_ contamination can impair the innate immunity of the small intestine by suppressing these receptors. Therefore, AFB_1_ negatively modulates the mucosa-associated microbiota, induces microbial-sensing receptors, and directly impairs the intestinal immune system in animals.

**Table 1:** Intestinal microbiota, immune response and oxidative damage products, and intestinal barrier function in chickens and pigs fed diets contaminated with aflatoxin B1 (AFB_1_). Changes were indicated using ↑ (increase) and ↓ (decrease).

Age or IBW ^1^	Experimental Period (d)	AFB_1_ (µg/kg)	Result ^2,3^	Reference
Intestinal microbiota				
Chicken (d)				
1	42	42	Relative abundance (RA): ↑ *Staphylococcus xylosus* (jejunal tissue)	[42]
1	21	40	Cell count: ↑ *Escherichia coli*, ↑ *Clostridium perfringens*, and ↑ Gram-negative bacteria (ileal digesta)	[47]
1	42	600	Cell count: ↑ *Coliforms* (cecal digesta)	[48]
1	42	1000	↓ acetate, ↓ propionate, and ↓ butyrate (feces)	[49]
7	14	394 to 1574	Cell count: ↑ *Escherichia coli*, ↑ *Salmonella*, and ↑ *Klebsiella* (ileal digesta)	[22]
22	21	40	RA: ↑ *Staphylococcus*, ↑ *Escherichia-shigella*, ↓ *Lactobacillus*, ↓ *Burkholderia-caballeronia-paraburkholderia*, ↓ *Romboutsia*, and ↓ *Corynebacterium* (jejunal tissue)	[19]
Pig (kg)				
6	48	180	RA: ↓ *Lactobacillus kitasatonis* (jejunal mucosa)	[43]
38	102	102	Cell count: ↑ *Escherichia coli* (colonic digesta)	[26]
Immune response and oxidative damage products				
Chicken (d)				
1	7 to 21	300	↓ IgA^+^ number and ↓ sIgA, ↓ IgA, and ↓ IgG (ileum)	[50]
1	7 to 21	600	mRNA expression: ↑ TNF-α, ↑ CASPASE-3, ↑ CASPASE-8, and ↑ CASPASE-10 (jejunum)	[51]
1	7 to 21	600	mRNA expression: ↓ TLR2-2, ↓ TLR4, and ↓ TLR7 (small intestine)	[46]
1	21	600	↓ IgA^+^ number and mRNA expression: ↓ IgA, ↓ pIgR, ↓ IgM, and ↓ IgG (small intestine)	[52]
1	21	100	↑ diamine oxidase and ↑ endotoxin (small intestine)	[53]
1	21	2000	↓ IgA (small intestine)	[54]
1	42	1000	↓ sIgA, ↑ IL-1β, and ↑ TNF-α (ileum)	[49]
22	21	40	mRNA expression: ↑ TLR2, ↑ NOD1, ↑NF-κB, ↑ iNOS, ↑ IL-6, ↑ IL-8, and ↑ TNF-α (jejunum)	[19]
Pig (kg)				
6	48	180	↑ IgA and ↑ protein carbonyl (jejunal mucosa)	[43]
56	35	180	↑ IgG (duodenal and jejunal mucosa)	
7	31	180	↑ protein carbonyl and ↑ TNF-α (jejunal mucosa)	[45]
7	30	500	↓ nitric oxide (small intestine)	[55]
9	30	320	↓ IFN-γ, ↓ IL-1β, ↓ TNF-α, ↓ IL-6, ↓ CAT, ↓ GPx, and ↓ SOD (duodenum), ↑ IFN-γ, ↑ IL-1β, and ↑ IL-6 (colon), and ↑ TBARS (duodenum and colon)	[56]
38	102	102	mRNA expression: ↑ TNF-α and ↑ IL-1β and ↑ TGF-β and ↓ SOD (jejunal mucosa)	[26]
Intestinal barrier function				
Chicken (d)				
1	7 to 21	600	↓ goblet cells (small intestine)	[46]
1	21	40	mRNA expression: ↓ claudin-1, ↓ sIgA, and ↓ pIgR (jejunum)	[47]
1	42	1000	mRNA expression: ↓ occludin, ↓ claudin-1, and ↓ zonula occludens-1 (ileal mucosa)	[49]
1	42	200	mRNA expression: ↓ claudin-3, ↓ occludin, and ↑ claudin-2 (jejunum)	[57]
22	21	40	mRNA expression: ↑ CASPASE-3 (jejunum)	[19]
Pig (kg)				
38	102	102	mRNA expression: ↓ zonula occludens-1 (jejunal mucosa)	[26]
Not available ^4^	12 h	10 to 50	mRNA expression: ↓ CASPASE-3, ↑ zonula occludens-1, and ↑ occludin at 10 µg/kg of AFB_1_, mRNA expression: ↓ mucin 2 at 20 µg/kg of AFB, and mRNA expression: ↑ Bcl-2 and ↑ratio of Bax to Bcl-2 at 30 to 50 µg/kg of AFB_1_ (jejunal cell culture)	[24]
Not available ^4^	48 h	40	mRNA expression: ↓ Bcl-2, ↓ zonula occludens-1, ↑ BaX, ↑IL-6, and ↑ CASPASE-3 (jejunal cell culture)	[25]

^1^ IBW = initial body weight. ^2^ Result description based on the comparison between diets contaminated with AFB_1_ and the control diet. ^3^ IgA = immunoglobulin A; pIgR = polymeric immunoglobulin receptor; IgM = immunoglobulin M; IgG = immunoglobulin G; TLR2-2 = toll-like receptor 2 type-2; TLR4 = toll-like receptor 4; TLR7 = toll-like receptor 7; sIgA = secretory immunoglobulin A; IgG = immunoglobulin G; IL-1β = interleukin-1β; TNF-α = tumor necrosis factor-alpha; CASPASE-3 = cysteine-aspartic acid protease-3; CASPASE-8 = cysteine-aspartic acid protease-8; CASPASE-10 = cysteine-aspartic acid protease-10; IFN-γ = interferon gamma; IL-6 = interleukin-6; SOD = superoxide dismutase; CAT = catalase; GPx = glutathione peroxidases; TBARS = thiobarbituric acid reactive substances; TGF-β = transforming growth factor-beta; Bcl-2 = B-cell lymphoma protein 2; Bax = Bcl-2-associated X protein. ^4^ In vitro, porcine jejunal epithelial cells were used.

## 3. Influence of AFB_1_ on the Intestinal Health Parameters of Chicken and Pig

### 3.1. Immune Response and Oxidative Damage Products in the Small Intestine

The intestinal mucosa acts as a physical barrier between the body and the external environment [17]. Aflatoxin B1 enters the body through the intestinal mucosa, primarily in the small intestine due to it being a site of high absorption [21], meaning the enterocytes are highly exposed to these external compounds [58]. Aflatoxin B1 is a major contaminant in feedstuffs, and enterocyte exposure to AFB_1_ has negative impacts, impairing innate immunity in the small intestine of animals [18]. The impacts of AFB_1_ on the jejunal immune system can be seen via changes in the expression of immunoglobulins (Igs), representing innate immune status. In the small intestine of chickens, AFB_1_ decreases IgA^+^ cell numbers and reduces the expression of immunoglobulin-related genes, including IgA, pIgR, IgM, and IgG, in the duodenum, jejunum, and ileum, thereby impacting immune function [52]. Furthermore, AFB_1_ exposure reduces antibody production from certain immune cells, including IgA, IgG, and IgM [47] and also reduces secretory IgA levels in the jejunum of chickens [54].

Aflatoxin B1 also induces the production of pro-inflammatory cytokines and oxidative damage products in the jejunum. A previous study reported that aflatoxin exposure at 1000 µg/kg AFB_1_ for 42 d increased the levels of IL-1β and TNF-α in the small intestine of chickens [49]. In contrast, aflatoxin exposure at 500 µg/kg for 30 d decreased interferon-gamma (IFN-γ), interleukin-1β (IL-1β), TNF-α, and IL-6 levels in the small intestine of nursery pigs, whereas in the colon and liver, inflammatory cytokines were increased [55]. A possible explanation for the reduction in pro-inflammatory cytokines in the jejunum in pigs is likely due to high AFB_1_ exposure, which induces epithelial cells apoptosis and subsequently impairs the function of the immune system [24,46]. Aflatoxin B1 exposure also increases diamine oxidase and endotoxin levels in the intestine of chickens, which act as important biomarkers of intestinal health [53]. Similarly, this immune suppression caused by AFB_1_ also increases oxidative damage in the intestine of pigs, as evidenced by increased protein carbonyl levels in the jejunum [45] and elevated thiobarbituric acid-reactive substances (TBARSs) levels in intestinal tissues of pigs [56]. Additionally, AFB_1_ exposure affects antioxidant enzymes in the intestine, such as superoxide dismutase (SOD), catalase (CAT), and glutathione peroxidase (GPx) [56]. Aflatoxin B1 exposure increases SOD levels in the jejunal mucosa of pigs [26]. Thus, AFB_1_ exposure induces intestinal inflammation, disrupts immune responses, increases oxidative damage products, and impairs antioxidant activity in the small intestine, all of which can negatively impact intestinal health parameters related to the growth of animals.

### 3.2. Tight Junction Protein, Intestinal Morphology, Tissue Repair, and Nutrient Digestion

Aflatoxin B1 negatively impacts the viability of intestinal cells in animals [24] by inhibiting intestinal development through mechanisms such as G_2_/M cell cycle arrest in epithelial cells [59], reducing the number of goblet cells [46], and increasing apoptosis rates, as indicated by a rise in TUNEL-positive cells in the jejunum [51]. A possible mechanism for these impacts on intestinal cells is that AFB_1_ induces oxidative stress by generating reactive oxygen species (ROS), which damage cellular components, including lipid, protein, and DNA [60,61]. This oxidative damage disrupts cellular integrity and triggers apoptosis, or programmed cell death, resulting in the loss of vital epithelial cells in the intestinal lining [62,63]. Aflatoxin B1 also compromises intestinal permeability by reducing the relative gene expression of tight junction proteins, such as claudins, occludin, and zonula occludens, increasing lactate dehydrogenase activity in enterocytes, and decreasing trans-epithelial electrical resistance (TEER) in the small intestine [24,64]. The TEER directly correlates to tight junction integrity in the intestinal epithelium, indicating that AFB_1_ can more easily enter circulation when TEER is reduced [64]. Aflatoxin B1 also induces apoptosis in intestinal cells by disrupting the balance of key apoptotic markers, such as B-cell lymphoma protein 2 (Bcl-2), Bcl-2-associatetd X protein (Bax), and cysteine-aspartic acid protease-3 (CASPASE-3) [24]. Aflatoxin B1 increases Bax, a protein that promotes cell death by making the mitochondrial membrane more permeable and releasing apoptotic signals in jejunal cells of pigs, whereas AFB_1_ reduces Bcl-2, a protein that protects cells from dying, thereby weakening the cellular defense against apoptosis [25]. As a result, AFB_1_ increases in the Bax/Bcl-2 ratio, inducing apoptosis of intestinal cells [65]. Aflatoxin B1 also activates CASPASE-3, a crucial executioner enzyme in the apoptotic pathway, which cleaves essential cellular components, leading to the controlled cell breakdown and death of enterocytes in animals [24,65]. In addition, AFB_1_ disrupts the cell cycle in intestinal epithelial cells, causing G_2_/M phase arrest [59], which limits normal proliferation and regeneration. Increased intestinal permeability further exacerbates the negative impacts of AFB_1_, as it facilitates entry into the body. This damage to intestinal integrity leads to impaired intestinal morphology and reduced nutrient utilization, and ultimately, a decline in animal growth performance [66]. Consequently, the adverse impacts of AFB_1_ are pronounced under conditions of increased intestinal susceptibility [21].

Aflatoxin B1 impairs intestinal morphology, reducing villus height and increasing crypt depth, which reduces the villus height-to-crypt depth ratio (VH:CD), an indicator of impaired intestinal structure and nutrient absorption function (Table 2). This impaired morphology reduces nutrient digestion and absorption, resulting in malnutrition and consequently compromised growth performance (Table 3 and Table 4). Studies have shown that AFB_1_ exposure decreases both apparent the ileal digestibility (AID) and total tract digestibility (ATTD) of nutrients in chickens and pigs [26,27,45,67]. The small and large intestines have distinct physiological roles, with most nutrient absorption occurring in the small intestine, whereas water and other metabolites are absorbed in the large intestine of animals [68]. Aflatoxin B1 is primarily absorbed in the small intestine, similarly to nutrients, meaning their impacts could be more pronounced in the small intestine, whereas AFB_1_ residues in the large intestine mainly result from bile secretion after absorption in the small intestine [21]; therefore, the negative impacts of aflatoxins are typically more concentrated in the small intestine of animals. Aflatoxin B1, in particular, is the most toxic aflatoxin due to its high absorption rate, which is influenced by its small size and lipid solubility, allowing it to readily cross cell membranes in the small intestine [69,70]. Altogether, the negative impacts of AFB_1_ are major contributors to compromised intestinal morphology, tight junction integrity, and nutrient digestion and absorption. Collectively, aflatoxins disrupt the luminal and mucosal microbiota, particularly in the small intestine, trigger immune responses and oxidative damage, compromise intestinal morphology, and impair nutrient utilization, ultimately affecting animal growth.

### 3.3. Impacts of AFB_1_ After Absorption on Overall Health and Growth Performance

After absorption, AFB_1_ is partially metabolized in hepatic cells into an active form that binds to hepatic macromolecules, a process believed to contribute to its toxicity and carcinogenicity [75,76]. Upon entering the liver, AFB_1_ undergoes metabolic activation, primarily by cytochrome P450 enzymes, particularly CYP1A2 and CYP3A4 [57]. This bioactivation converts AFB_1_ into the highly reactive AFB_1_-8,9-epoxide, which can form adducts with DNA, proteins, and other macromolecules, leading to hepatotoxicity and carcinogenicity [77,78]. The binding of AFB_1_-8,9-epoxide to hepatic macromolecules disrupts normal cellular functions, adversely affecting immune function and enzyme activities [79,80], and increasing hepatotoxicity [81]. This hepatotoxicity often results in liver enlargement (hepatomegaly) as the liver attempts to counteract the damage [27,82]. Liver weights were increased with high AFB_1_ doses, as observed in experiments with doses ranging from 100 to 2500 µg/kg in feeds [27,82,83]. When the AFB_1_ detoxification capacity of the liver is overwhelmed, systemic impacts such as immunosuppression, metabolic dysfunction, and reduced growth performance in animals occur, as documented in literature reviews [84,85]. Additionally, AFB_1_ exposure compromises cell membrane integrity, leading to increased lipid peroxidation and elevated levels of malondialdehyde (MDA), a marker of oxidative stress. Studies have reported increased MDA levels in the livers of chickens exposed to AFB_1_, indicating oxidative damage and cell death [83,86,87,88]. The remaining AFB_1_ is converted into less toxic forms, which are then transported from hepatic cells into the blood and bile, along with un-metabolized parent compounds, and eventually are excreted in feces and urine [21,89].

**Figure 1:**
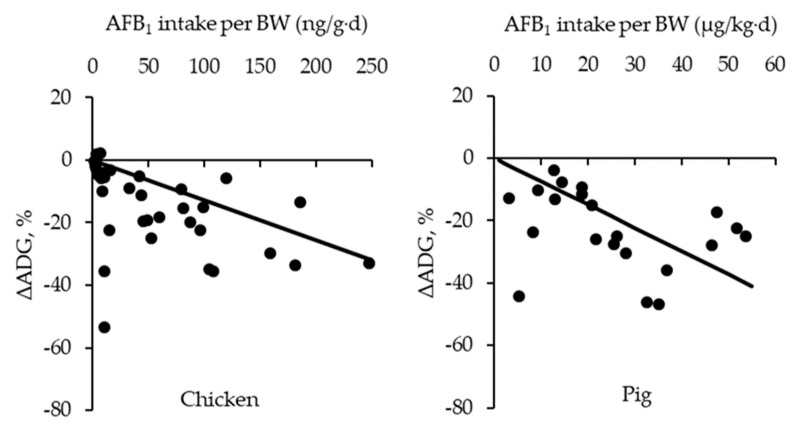
Change in average daily gain (ΔADG) of chickens and pigs fed diets contaminated with aflatoxin B1 (AFB_1_). The meta-analysis was conducted by Proc REG using the data from 27 peer-reviewed papers on chickens and 13 peer-reviewed papers on pigs to determine the impacts of AFB_1_ intake per body weight (BW) on ΔADG of animals. The equations were as follows: for chickens fed diets with AFB_1_ (solid line, ●): ΔADG (%)_chicken_ = −0.13 × AFB_1_ intake per BW (ng/g·d) with standard error of slope = 0.02, r^2^ = 0.48, and *p* < 0.01; and for pigs fed diets with AFB_1_ (solid line, ●): ΔADG (%)_pig_ = −0.74 × AFB_1_ intake per BW (µg/kg·d), with standard error of the slope = 0.11, r^2^ = 0.70, and *p* < 0.01. The AFB_1_ intake per BW ranged from 1.2 to 350.9 ng/g·d in chickens and 3.0 to 53.7 µg/kg·d in pigs.

To determine the negative impacts of AFB_1_ intake per BW on the % change in the average daily gain (ADG) of animals, a meta-analysis was conducted (Figure 1). The reason for using AFB_1_ intake per BW as the independent variable for the meta-analysis is because this approach can accurately determine the impacts of AFB_1_ on growth, as the trials involved animals with varying feed consumptions and body weights. The change in ADG relative to the control diet group was calculated as follows:

∆ADG (%) = (ADG of AFB_1_ treatment group−ADG of control group)/ADG of control group × 100

A literature search was conducted using the databases of PubMed and Google Scholar with keywords including aflatoxins, intestinal health, growth performance, and chickens and pigs, followed by screening after reading each article. The initial body weight (IBW) and experimental period ranges for chickens were 40 to 850 g and 7 to 62 d, respectively (Table 3), whereas the IBW and experimental period ranges for pigs were 7 to 50 kg and 21 to 90 d, respectively (Table 4). The AFB_1_ intake per body weight (BW) ranged from 1.2 to 350.9 ng/g·d in chickens and 3.0 to 53.7 µg/kg·d in pigs. Data on AFB_1_ co-contaminated with other mycotoxins were excluded to determine the impact of AFB_1_ on the ADG of animals. The AFB_1_ intake per BW of animals in each research article was calculated by multiplying the dietary AFB_1_ content with the overall average daily feed intake of each treatment divided by the mean BW of animals. In the meta-analysis, the impact of AFB_1_ intake per BW of animals on % change in body weight gain was evaluated using a linear regression with the Proc REG procedure in SAS (SAS Inst. Inc., Cary, NC, USA). The independent variable was the AFB_1_ per BW (ng/kg·d for chicken and µg/kg·d for pig), and the dependent variable was the calculated % change in ADG (∆ADG, %) relative to the control group. To ensure the regression model passed through the origin, the NOINT option was applied, forcing the intercept of the regression line to be zero. This approach assumes that in the absence of AFB_1_ intake, there would be no impact on ADG (∆ADG = 0%). Based on the meta-analysis, the equations for chicken and pig in diets containing AFB_1_ were as follows (Figure 1):

For chicken: ΔADG (%)_chicken_ = −0.13 × AFB_1_ intake per BW (ng/g·d)

For pig: ΔADG (%)_pig_ = −0.74 × AFB_1_ intake per BW (µg/kg·d).

These equations indicate that for every 1 µg/kg·d of AFB_1_ intake per BW, ADG decreases by 0.13% in chickens and by 0.74% in pigs. For example, AFB_1_-contaminated diets (300 µg/kg) were fed to nursery pigs (weighing 7 to 25 kg, averaging 16 kg) with an ADFI of 0.50 kg, estimating a 6.9% reduction in ADG compared to the ADG of pigs fed non-contaminated diets.

Studies report that AFB_1_ absorption is higher in early life stages compared to older life stage in rats [21]. This increased absorption is likely due to an immature digestive system, differences in lipid composition of the epithelial cell membrane in the small intestine at different ages, and age-related changes in liver metabolic activity that may also have impacts of AFB_1_ on animal growth [90,91]. Additionally, in pigs, the weaning period could leave them more susceptible to AFB_1_ exposure due to the greater likelihood of opportunistic pathogenic bacterial colonization in the intestine, triggered by dietary challenges and reduced feed intake. Considering the collective findings on the negative impacts of AFB_1_ on intestinal microbiota, intestinal health, overall health, and growth performance of animals, reducing AFB_1_ absorption could be a key factor in mitigating its adverse impacts on animals.

**Table 3:** Growth performance of chickens fed diets contaminated with aflatoxin B1 (AFB_1_) and AFB_1_ co-contaminated with other mycotoxins ^1,2^.

IBW (g) ^3^	Experimental Period (d)	Dietary AFB_1_ (µg/kg) ^4^	AFB_1_ Intake per BW (ng/g·d) ^5^	Contamination Type	Growth Performance (% Change)			Reference
					ADG	ADFI	G:F	
40	44	25	2.0	Natural	−3.17 **	−2.96 **	−0.22	[88]
40	21	40	5.1	Natural	−7.39 **	−5.32 **	−2.19 **	[92]
40	35	1000	98.7	Culture	−14.99 **	−4.34	−11.14 **	[74]
33	35	1000	119.7	Not available	−5.69 **	4.65	−9.88 **	[93]
35	62	15	1.2	Culture	−0.32	−0.13	−0.19	[94]
		30	2.3		−1.92	−1.29	−0.64	
		45	3.5		−2.88	−1.03	−1.87 **	
		60	4.7		−4.79	−2.83	−2.02 **	
35	21	100	15.2	Pure AFB_1_ with LPS (1.7 × 10^6^ EU/bird)	−22.44 **	−13.80 **	−10.02 **	[53]
40	28	1000	104.3	Culture	−34.74 **	−28.93 **	−8.17 **	[83]
40	42	500	52.6	Culture	−25.00 **	−7.62 **	−18.81 **	[95]
40	42	100	10.7	Culture	−35.56 **	−26.50 **	−12.31 **	[96]
40	35	1000	108.3	Culture	−35.33 **	−26.29 **	−12.54 **	[97]
40	21	2500	350.9	Culture	−16.77 **	−14.77 **	−2.35	[98]
40	49	500	41.4	Culture	−5.28	−0.31	−4.98	[99]
		1000	87.6		−19.90 **	−9.83 **	−11.17 **	
		2000	181.6		−33.73 **	−21.45 **	−15.64 **	
40	42	100	8.6	Pure AFB_1_	−9.78 **	−5.69	−4.34 **	[100]
40	23	750	79.6	Culture	−9.42 **	−9.67 **	0.27	[101]
		1500	158.9		−29.70 **	−28.25 **	−2.02 **	
40	49	129	10.2	Pure AFB_1_	−5.53 **	5.41	−10.37 **	[102]
		385	32.7		−8.91 **	9.67 **	−16.95 **	
		895	96.8		−22.48 **	19.57 **	−35.17 **	
40	42	50	4.1	Pure AFB_1_	−4.05 **	−9.67 **	6.23 **	[103]
		100	7.9		−5.74	−14.77	10.60	
40	42	500	45.3	Culture	−19.44	−16.17	−3.91	[104]
40	42	200	15.7	Pure AFB_1_	−3.29 **	−1.46	−1.82 **	[57]
40	20	1500	185.4	Culture	−13.51 **	−8.20 **	−6.88 **	[71]
42	37	40	3.2	Culture	1.96	3.95 **	−1.91 **	[105]
43	42	500	43.4	Culture	−11.29 **	−1.88	−9.6	[106]
43	42	600	48.7	Culture	−19.23 **	−9.85 **	−10.40 **	[48]
45	21	60	6.6	Pure AFB_1_	2.07	2.02	0.05	[72]
833	21	120	11.0		−4.00	−1.43	−2.61	
47	42	1000	80.8	Pure AFB_1_	−15.47 **	−6.94	−9.16 **	[82]
48	21	2000	269.6		−25.12 **	3.33	−27.54 **	[54]
838	7	100	10.4	Pure AFB_1_	−53.33 **	−4.76 **	−51.00 **	[87]
140	14	394	60.0	Culture	−18.34 **	−7.17 **	−12.04 **	[22]
		1574	247.5		−32.87 **	−14.88 **	−21.14 **	
				Mycotoxin ^6^, µg/kg				
45	42	25	1.7	DON: 1000, ZEA: 90, and OTA: 90	−4.10 **	−4.85 **	0.78	[107]
		50	3.3	DON: 1000, ZEA: 90, and OTA: 475	−6.15 **	−8.13 **	2.16	
50	21	42	4.9	DON: 86 and ZEA: 50 µg/kg	−9.11 **	−15.71 **	7.83 **	[42]
	42	42	4.3	DON: 86 and ZEA: 50 µg/kg	−8.80	3.67	−12.02	
40	49	500	38.1	OTA: 1000	−20.22 **	−21.83 **	2.06	[99]
		1000	82.0	OTA: 2000	−35.41 **	−30.69 **	−6.81	
		2000	194.1	OTA: 4000	−51.00 **	−35.98 **	−23.47 **	
162	34	330	30.2	ZEA: 4, AFB_2_: 80, AFG_1_: 30, and AFG_2_: 7	−4.52 **	−2.73	−1.84 **	[108]
45	35	20	1.9	AFB_2_: 5, AFG_1_: 10, and AFG_2_: 4	−8.42 **	−2.43	−6.14 **	[109]
40	42	100	8.4	DON: 2000, ZEA: 280, and FMN: 5800	−8.04	−0.66	−7.43	[110]

^1^ Asterisk marks (**) represent statistical tendency (*p* < 0.10) and significant difference (*p* < 0.05), respectively. ^2^ The percentage increase or decrease in the average daily gain (ADG), average daily feed intake (ADFI), and gain-to-feed ratio (G:F) was determined in aflatoxin groups relative to the control group. ^3^ References with multiple BWs indicate multiple levels or studies within the same publication. ^4^ Total AFB_1_ content per kg of the diet. ^5^ Average AFB_1_ consumed per g of BW, calculated by multiplying the AFB_1_ content of the diet (ng/g) by the ADFI (g/d), divided by the mean BW of animals (g). ^6^ DON = deoxynivalenol; ZEA = zearalenone; OTA = ochratoxin; AF = aflatoxin.

**Table 4:** Growth performance of pigs fed diets contaminated with aflatoxin B1 (AFB_1_) and AFB_1_ co-contaminated with other mycotoxins ^1,2^.

IBW (kg) ^3^	Experimental Period (d)	Dietary AFB_1_ (µg/kg) ^4^	AFB_1_ Intake/BW (µg/kg·d) ^5^	Contamination Type	Growth Performance (% Change)			Reference
					ADG	ADFI	G:F	
7	28	182	9.2	Natural	−10.20 **	−8.43 **	−1.95 **	[111]
7	28	182	12.8		−3.98 **	−5.56	1.65	
7	40	500	20.9	Natural	−15.17 **	−15.48 **	0.37	[112]
9	35	922	51.8	Natural	−22.38 **	−20.00 **	−2.97	[113]
9	30	320	-	Natural	−45.31 **	-	-	[56]
11	28	420	18.6	Natural	−11.54 **	−15.93 **	4.35	[114]
		840	32.6		−46.15 **	−40.71 **	−19.57	
9	42	800	36.9		−35.94 **	−37.88 **	2.04	
11	28	800	53.7	Natural	−25.00 **	−11.36	−15.38 **	[113]
10	33	500	28.1		−30.30 **	−31.21 **	1.31	
10	28	800	48. 0		−17.46 **	−20.93 **	4.39	
53	66	385	12.9	Natural	−12.99 **	−11.85 **	−1.29	[114]
		750	21.7		−25.97 **	−25.09 **	−1.18	
		1480	35.2		−46.75 **	−43.90 **	−5.08 **	
9	42	373	18.6	Culture	−9.26	−10.09	1.01	[115]
9	21	500	25.5	Culture	−27.52 **	−29.19 **	2.35	[116]
10 ^6^	35	250	14.4	Culture	−7.64 **	−14.61 **	8.17 **	[117]
		500	26.2		−25.04 **	−29.38 **	6.15 **	
		800	46.4		−28.01 **	−25.74 **	−3.05	
11	41	200	8.3	Culture	−23.64 **	−10.00	−15.06 **	[118]
11	30	140	-	Culture	−7.36	-	-	[119]
		280	-		−33.33 **	-	-	
12	28	385	-	Purified AFB_1_	−14.35	-	-	[120]
		867	-		−36.14	-	-	
		1807	-		−75.33 **	-	-	
16	21	110	5.3	Culture	−44.35 **	−33.81 **	−19.75 **	[77]
16	28	2500	-	Culture	−54.55 **	-	-	[121]
30	90	110	3.0	Culture	−12.90 **	−6.37	−7.09 **	[122]
				Mycotoxin ^7^, µg/kg				
6	35	20	1.1	FMN: 1600	2.26	1.77	0.48	[123]
6	32	217	8.2	FMN: 7506	−17.35 **	−19.28 **	2.39 **	[66]
7	31	180	7.9	DON: 2000	−16.47 **	−20.62 **	5.18 **	[45]
10	21	180	8.3	DON: 2000	−13.05 **	−13.54 **	0.58 **	
9	42	150	6.8	DON: 1100	−10.87 **	−6.99	−5.53	[124]
14	33	64	2.9	DON: 320 and FMN: 42	−11.54	−6.73	−5.15	[125]
		124	5.4	DON: 548 and FMN: 84	−17.31 **	−5.15 **	−1.44	
		182	7.8	DON: 768 and FMN:128	−21.15 **	−4.35 *	−0.32	
29	26	190	8.9	FMN: 8000	−6.96 **	−9.88 **	3.09	[18]
38	102	286	8.2	ZEA: 50 and DON: 406	−8.63 **	−8.33 **	−0.32	[26]
6	48	180	5.9	FMN: 9000 and DON: 1000	−15.79 **	−18.48 **	2.88	[43]
56	35	180	6.7	FMN: 14000	−5.96	−6.25	0.26	

^1^ Asterisk marks (*, **) represent statistical tendency (*p* < 0.10) and significant difference (*p* < 0.05), respectively. ^2^ The percentage increase or decrease in the average daily gain (ADG), average daily feed intake (ADFI), and gain-to-feed ratio (G:F) was determined in aflatoxin groups relative to the control group. ^3^ IBW = initial body weight; References with multiple BWs indicate multiple levels or studies within the same publication. ^4^ Total AFB_1_ content per kg of the diet. ^5^ Average AFB_1_ consumed per kg of BW, calculated by multiplying the AFB_1_ content of the diet (µg/kg) by the ADFI (kg/d), divided by the mean BW of animals (kg). ^6^ The presence of aflatoxin linearly decreased (*p* < 0.05) ADG, ADFI, and G:F of nursery pigs and had a quadratic effect (*p* < 0.05) on G:F. ^7^ DON = deoxynivalenol; ZEA = zearalenone; FMN = fumonisin.

## 4. Aflatoxin B1 Mitigation Strategies for Intestinal Health and Growth Performance of Monogastric Animals

Mycotoxin-detoxifying agent is a collective term including non-nutritive feed additives that specifically target mycotoxins for their detoxification [5,126]. Mycotoxin-detoxifying agents are categorized into mycotoxin-adsorbing agents and mycotoxin-biotransforming agents. These feed additives have been used individually, whereas multiple additives have been used together for enhanced detoxification efficacy and are referred to as multi-component mycotoxin-detoxifying agents. Good examples of multi-component mycotoxin-detoxifying agents include a combinational use of clay and yeast cell wall [123,127], and clay and enzymes [66,128]. Other feed additives have also been used to help animals to cope with mycotoxins by improving intestinal barrier functions and anti-oxidative capacity [72,129,130].

### 4.1. Mycotoxin-Adsorbing Agent

Mycotoxin-adsorbing agents are extensively supplemented in monogastric animal feeds to mitigate the negative impacts of aflatoxins (Table 5) [31,113,114]. The mode of action for mycotoxin-adsorbing agents relies on the characteristics of the agents, which bind the toxins and excrete them along with undigested waste [131,132]. Inorganic compounds, such as clay and minerals, are naturally occurring or modified aluminosilicates. Aluminosilicates include bentonites, montmorillonites, kaolinites, and zeolite, characterized by their layered structure and high cation-exchange capacity [133,134]. These inorganic compounds adsorb toxins through ionic and hydrophobic interactions, preventing their absorption in the GIT [131], thereby reducing its toxicity and improving intestinal health and growth performance in animals [47,56]. In vitro trials evaluating the efficiency of clay compounds in adsorbing AFB_1_ reported high adsorption rates, ranging from 92 to 99% [131,132,135], indicating that clay compounds have a high affinity for AFB_1_. Charcoal can also be used to adsorb multiple mycotoxins as a mycotoxin-adsorbing agent; however, activated charcoal also adsorbs essential nutrients in feed [136]. Notably, the clay compounds show less interference in the utilization of vitamins or micro-minerals in the GIT of animals, making clay compounds commonly utilized to prevent the impacts of AFB_1_ on intestinal health and growth of animals. For other mycotoxins, such as deoxynivalenol (DON) and zearalenone (ZEA), the adsorption efficacy of clay compounds is relatively low and highly variable among different adsorbing agents when compared to the adsorption efficiency for AFB_1_ [131]. The reason for the high adsorption of AFB_1_ by clay may be due to the hydrophobic characteristics of AFB_1_, causing strong ionic and electrostatic interactions with clay [131]. Aflatoxin B1 also has a large, planar, and rigid aromatic structure [137], allowing it to fit closely with the flat, layered surfaces of clays like montmorillonite [131].

In the jejunum of chickens, the inorganic compound, hydrated sodium calcium aluminosilicate (HSCA), decreased *Escherichia coli* and gram-negative bacteria in the ileum and increased the relative mRNA expression of claudin-1, secretory IgA, and pIgR [47]. Also, the inorganic compound, 0.40% bentonite, increased intestinal morphology and increased the nutrient digestion of chickens [27]. The beneficial effects of clay compounds also improved the growth performance of animals (Table 6). Thus, clay compounds effectively adsorb AFB_1_ in the digesta of animals, thereby mitigating its negative impacts on intestinal health, which are highly related to growth performance. The clay compound, however, could reduce feed intake if the clay compound was supplemented to chicken feeds at a rate of 1% [96], potentially indicating that high doses of clay compounds could reduce feed palatability.

Yeast cell wall compounds, another type of mycotoxin-adsorbing agent, can efficiently adsorb AFB_1_ due to the manno-oligosaccharides and β-glucan structures present in their composition [138,139]. Specifically, β-glucan in the yeast cell wall has high adsorbability for AFB_1_ in the digesta of pigs, with AFB_1_ detoxification by *Saccharomyces cerevisiae* strains reaching 65% after 24 h of incubation [140]. The yeast cell wall also has a prebiotic effect on intestinal health and growth by positively modulating immune function through jejunal dectin-1 activation [141,142], which could mitigate the negative impacts of AFB_1_ on the intestinal health of animals [139]. A previous study reported that yeast cell wall supplementation in feed contaminated with 180 µg/kg of AFB_1_ decreased jejunal IgA and protein carbonyl, increased jejunal villus height and crypt cell proliferation, and improved AID of DM and CP in nursery pigs [43]. Manno-oligosaccharide supplementation in feeds, a compound found in high concentrations in yeast cell walls, can mitigate the negative impacts of AFB_1_ on the intestinal health and growth of animals, decreasing the *Escherichia coli*, *Salmonella*, *Klebsiella*, and total gram-negative bacteria counts in the ileum and improving intestinal morphology including villus height, crypt depth, and VH:CD [22]. Additionally, the detoxification mechanism of *Lactobacillus*, *Bifidobacterium*, and *Enterococcus* generally involves binding AFB_1_, which helps reduce the negative impacts of AFB_1_ on the intestinal health of animals [143,144].

**Table 5:** Intestinal health of chickens and pigs fed diets contaminated with aflatoxin B1 (AFB_1_) including mycotoxin-detoxifying agents or other feed additives. Changes were indicated using ↑ (increase) and ↓ (decrease).

Age or IBW ^1^	Experimental Period (d)	AFB_1_ (µg/kg)	Type	Level (%)	Result ^2^	Reference
Mycotoxin-adsorbing agent						
Chicken (d)						
1	21	40	Clay (HSCA ^3^)	0.30	Cell count: ↓ *Escherichia coli* and ↓ Gram-negative bacteria (ileal digesta) and mRNA expression: ↑ claudin-1, ↑ sIgA, and ↑ pIgR (jejunum)	[47]
11	19	250	Clay (bentonite)	0.40	↑ villus height and ↑ villus surface area (ileum) and ↑ ATTD of CP and EE and AME	[27]
			Mineral (zeolite)	0.40	↑ ATTD of CP	
7	21	394 to 1574	Manno-oligosaccharides	0.10 to 0.20	Cell count: ↓ *Escherichia coli*, ↓ *Salmonella*, *Klebsiella*, and ↓ Gram-negative bacteria (ileal digesta) and ↑ villus height, ↑ VH:CD, ↓ crypt depth, and ↓ goblet cell counts (jejunum)	[22]
	35	394 to 1574	Manno-oligosaccharides	0.10 to 0.20	Cell count: ↓ *Escherichia coli*, ↓ *Salmonella*, and ↓ *Klebsiella* (ileal digesta) and ↑ villus height, ↑ crypt depth, ↑ VH:CD, and ↓ goblet cell counts (jejunum)	
1	21	2000	Cellulosic polymer	0.30	↓ relative weight of intestine	[54]
1	42	1000	*Lactobacillus salivarius*	10^8^ CFU/kg	↑ acetate, ↑ propionate, and ↑ butyrate (feces) and ↓ IL-1β and ↓ TNF-alpha (ileum)	[49]
1	21	40	*Lactobacillus acidophilus*, *Lactobacillus plantarum*, and *Enterococcus faecium*	3 × 10^10^ CFU/kg	Cell count: ↓ *Clostridium perfringens*, ↓ *Escherichia coli*, and ↓ Gram-negative bacteria (ileal digesta) and mRNA expression: ↑ claudin-1, ↑ sIgA, and ↑ pIgR (jejunum) and ↓ visceral lesion score (small intestine)	[47]
Pig (kg)						
6	48	180	Yeast cell wall	0.20	↓ IgA and ↓ protein carbonyl (jejunal mucosa) and ↑ AID of DM and CP	[43]
Multi-component mycotoxin-detoxifying agent						
Chicken (d)						
1	21 to 42	200	Clay (bentonite) + yeast cell wall	0.20	mRNA abundance: ↑ claudin-1 on d 21 and mRNA abundance: ↑ claudin-2 and ↑ occludin on d 42 (jejunum)	[57]
1	42	600	Adsorbing gent [clay (bentonite), activated charcoal, *Lactobacillus* sp., and *Bifidobacterium* sp.] + biotransforming agent (*Bacillus* sp.)	0.10	Cell count: ↓ *Coliforms* (cecal digesta)	[48]
1	21 to 42	42	Adsorbing gent ^4^ [clay (montmorillonite), *Lactobacillus casei*, and *Enterococcus faecalis*] + biotransforming agent ^5^ (*Bacillus subtilis*, *Candida utilis*, and mycotoxin-degrading enzyme)	0.10	↑ ATTD of CP	[42]
				0.15	↓ crypt depth, ↑ VH:CD, and ↑ relative jejunum weight on d 42 (jejunum) and ↑ ATTD of CP	
Pig (kg)						
7	30	500	Clay + yeast cell wall	0.10	↓ villus height (small intestine)	[55]
Not available ^6^	48 h	40	Adsorbing agent (*Lactobacillus casein* and *Candida utilis*) + biotransforming agent (*Aspergillus oryzae*, *Bacillus subtilis*, and mycotoxin-degrading enzyme)]	5.00	mRNA abundance: ↓ IL-6, ↑ occludin, ↑ ZO-1, ↓ Bax, ↓ CASPASE-3, and ↑ Bcl-2 and ↑ rate of cell viability, ↓ % of necrotic cell, ↓ early apoptotic cell, and ↓ viable cell rate (jejunal cell culture)	[25]
Other feed additive						
Chicken (d)						
1	34	250	Phytobiotics	0.03 to 0.05	↑ villus height, ↑ VH:CD, and ↓ crypt depth (jejunum)	[145]
1	42	600	Milk thistle (*Silybum marianum*)	1.00	Cell count: ↓ *Coliforms* (cecal digesta)	[48]
1	35	1000	Phytobiotics	0.05	No difference in the number of bacteria (cecal digesta) and ↑ villus height, ↑ VH:CD, and ↓ crypt depth (jejunum)	[74]
1	7 to 21	600	Selenium	0.4 mg/kg	↑ villus height, ↑ VH:CD, ↑ number of absorptive cells, ↓ crypt depth, and ↓ TUNEL-positive cells (jejunum)	[51]
1	14 to 21	300	Sodium selenite	0.4 mg/kg	↑ IgA^+^ number and ↑ IgG (ileum)	[50]
1	21	2000	Cellulosic polymer + curcumin	0.50	↑ IgA and ↓ relative weight of intestine (small intestine)	[54]
1	35	1000	Toxin binders (adsorbing agents + mycotoxin-degrading enzyme + plant extract)	0.05	No difference on the number of bacteria (cecal digesta) and ↑ villus height, ↑ VH:CD, and ↓ crypt depth (jejunum)	[74]
Pig (kg)						
9	30	320	Grape seed waste	8.00	↑ SOD, ↑ antioxidant capacity, ↑ thiobartituric acid reactive substances, ↑ IL-6, and ↑ IL-8 (duodenum) and ↑ CAT, ↑ GPx, ↓ IFN-γ, ↓ IL-1β, ↓ TNF-α, and ↓ IL-6 (colon)	[56]

^1^ IBW = initial body weight. ^2^ Comparison of the effects of mycotoxin mitigation agents in diets contaminated with AFB_1_, compared to the effects of diets contaminated with AFB_1_ without mycotoxin mitigation agents, in chickens and pigs; VH:CD = villus height-to-crypt depth ratio; SOD = superoxide dismutase; CAT = catalase; GPx = glutathione peroxidase; IL-6 = interleukin-6; IL-8 = interleukin-8; IFN-γ = interferon-gamma, IL-1β = interleukin-1β, TNF-α = tumor necrosis factor α; AID = apparent ileal digestibility; AME = apparent metabolizable energy; ATTD = apparent total tract digestibility; CP = crude protein. ^3^ HSCA = hydrated sodium calcium aluminosilicate. ^4^ Adsorbing agent = *Lactobacillus casei* (1.0 × 10^8^ CFU/g) and *Enterococcus faecalis* (1.0 × 10^10^ CFU/g) were included. ^5^ Biotransforming agent *= Bacillus subtilis* (1.0 × 10^8^ CFU/g) and *Candida utilis* (1.0 × 10^8^ CFU/g) were included. ^6^ In vitro, porcine jejunal epithelial cells were used.

**Table 6:** Growth performance of chickens and pigs fed diets contaminated with aflatoxin B1 (AFB_1_) including mycotoxin-adsorbing agent.

Age or IBW ^1^	Experimental Period (d)	AFB_1_ (µg/kg)	Type	Level (%)	Growth Performance ^2^ (% Change)						Reference
					ADG		ADFI		G:F		
					vs. AFB_1_^3^	vs. Control ^4^	vs. AFB_1_	vs. Control	vs. AFB_1_	vs. Control	
Chicken (d)											
1	21	40	Clay (HSCA ^4^)	0.30	4.47 **	−3.26 **	3.66 **	−1.85 **	0.78 **	−1.43 **	[92]
1	20	250	Clay (bentonite)	0.40	17.05 **	−6.36	6.88	−0.33	9.52 **	−6.05	[27]
			Clay (zeolite)	0.40	18.41 **	−5.27	10.26 **	2.83	7.40 **	−7.87 **	
1	42	100	Clay (sodium bentonite)	0.50	29.12 **	−16.70 **	16.21 **	−14.77 **	11.10 **	−2.26 **	[96]
			Clay (sodium bentonite)	1.00	4.85 **	−32.35 **	−1.91 **	−28.06 **	6.89 **	−5.96 **	
			Silicate (sorbatox)	0.50	4.08 **	−32.85 **	−2.42 **	−28.43 **	6.65 **	−6.17 **	
			Silicate (klinofeed)	0.20	3.12 **	−33.47 **	0.54 **	−26.27 **	2.56 **	−9.77 **	
1	42	500	Clay (smectite)	0.20	14.29 **	−14.29 **	0.00	−7.62 **	14.29 **	−7.22 **	[95]
1	42	1000	Diatomaceous earth extracted from a quarry	0.10	2.15 **	−13.62 **	2.29	−4.79	−0.14	−9.27 **	[82]
				0.20	18.84 **	0.50	11.87 **	4.13	6.23	−3.48	
				0.50	14.29	−3.35 **	6.77	−0.62	7.04 **	−2.74	
1	35	1000	Yeast cell wall	0.20	3.45 **	−31.82 **	3.45 **	−24.05 **	0.00	−10.23 **	[97]
7	14	500	Mannanoligosaccharides	0.10	4.35 **	2.33 **	1.98 **	−12.73 **	−5.38 **	−7.77 **	[22]
				0.20	10.87 **	6.98 **	3.64 **	−7.27 **	−1.08 **	−6.26 **	
		2000	Mannanoligosaccharides	0.10	13.16 **	3.70 **	9.12 **	−21.82 **	−9.68 **	−13.44 **	
				0.20	18.42 **	6.17 **	11.54 **	−18.18 **	−7.53 **	−11.52 **	
1	21	2000	Cellulosic polymer	0.30	25.15 **	−6.19 **	−3.23 **	0.12	21.56 **	−2.13 **	[54]
1	42	500	Mineral (toxin binder)	0.10	7.55 **	−5.00 **	1.98 **	0.00	5.46 **	−5.00 **	[106]
Pig (kg)											
9	35	922	Clay (sodium bentonite)	1.00	20.15 **	−6.73	21.59 **	−2.73	−1.18	−4.12	[113]
9	42	150	Clay (montmorillonite)	0.02	5.84	−5.67	4.22	−3.06	1.9	−2.69	[124]
9	42	500	Clay (sodium bentonite)	0.50	37.46 **	−0.41	9.43	2.96	25.62 **	−3.27	[146]
			Clay (montmorillonite) + zeolite	0.50	18.03 **	−14.49	−4.83	−10.45	24.01 **	−4.51	
			Clay (calcium bentonite)	0.50	22.54 **	−11.22	−5.16	−10.77	29.21 **	−0.51	
			Clay (attapulgite)	0.50	−0.56	−27.96	−8.98	−14.36	9.25	−15.88	
9	42	373	Clay (maifanite)	1.00	12.24	1.85	8.16	−2.75	3.77	4.73	[115]
10	21	500	Clay (HSCA ^5^)	0.50	36.20 **	−1.28	39.14 **	−1.47	−2.11	0.19	[116]
11	28	840	Clay (HSCA)	0.50	71.43 **	−7.69	74.63 **	3.54	−1.83	−10.85	[114]
11	28	800	Clay (calcium bentonite)	0.50	29.17 **	−3.13	13.68	0.76	13.63 **	−3.85	[113]
			Clay (HSCA)	0.50	20.83 **	−9.38	8.55	−3.79	11.32	−5.81	
			Clay mineral (palygorskite)	0.50	10.42	−17.19	0.00	−11.36	10.42	−6.57	
			Clay mineral (sepiolite)	0.50	25.00 **	−6.25	0.85	−10.61	23.94 **	4.87	
11	41	200	Clay (bentonite)	0.40	26.19 **	−3.64	7.41	−3.33	17.49 **	−0.31	[118]
				0.50	23.81 **	−5.45 **	11.11	0.00	11.43	−5.45	
20	21	1100	Clay (HSCA)	0.50	166.67 **	−18.99	38.82	−19.73	92.09 **	0.92	[77]
30	90	110	Clay (montmorillonite)	0.30	5.56	−8.06	5.44	−1.27	0.11	−6.88	[122]
			Clay (montmorillonite nanocomposite)	0.30	12.96 **	−1.61	7.48	0.64	5.10 **	−2.24	
			Clay (montmorillonite nanocomposite)	0.30	12.96 **	−1.61	7.54	0.73	5.04 **	−2.33	
6	35	20	Yeast cell wall	0.20	5.42 **	7.8	6.10 **	7.98	−0.64	−0.17	[123]

^1^ IBW = initial body weight. ^2^ Asterisk marks (**) represent statistical tendency (*p* < 0.10) and significant difference (*p* < 0.05), respectively. ^3^ The percentage increase or decrease in the average daily gain (ADG), average daily feed intake (ADFI), and gain-to-feed ratio (G:F) was determined in AFB_1_ contamination with mitigation agents relative to the aflatoxin contamination group. ^4^ The percentage increase or decrease in the ADG, ADFI, and G:F was determined in aflatoxin contamination with mitigation agents relative to the control group. ^5^ HSCA = hydrated sodium calcium aluminosilicate.

### 4.2. Mycotoxin-Biotransforming Agent

To mitigate the negative impacts of AFB_1_, biotransforming agents such as mycotoxin-degrading enzymes and specific bacteria that secrete enzymes that degrade mycotoxins into non-toxic metabolites can be supplemented in feeds to counteract its negative impacts on the intestinal health and growth of animals [42,47]. Selected strains of *Bacillus* spp. have generally demonstrated a high ability to degrade AFB_1_ into non-toxic metabolites [147]. A possible explanation is that these bacteria produce various enzymes, such as laccase and lactonase, which target AFB_1_ and convert it to aflatoxin Q1 [148] and aflatoxin D1 [149], respectively, both of which are less toxic metabolites [150,151]. *Bacillus cereus* and *Bacillus subtilis* are known to produce lactonase, which cleaves the lactone ring of AFB_1_ by approximately 40% and 50%, respectively, after 72 h of incubation in an in vitro study [152]. Previous studies also reported that *Bacillus TUBF1 B.* [153] and *Bacillus subtilis UTBSP1* [154] achieved over 80% AFB_1_ degradation after 72 h of incubation in in vitro studies. Additionally, supplementation with *Bacillus subtiltis* showed additional beneficial effects on the intestinal health of animals by modulating the intestinal microbiota, reducing immune responses [155], and improving the intestinal morphology of animals [156]. A previous study reported that *Nocardia corynebacteroides* showed 70% AFB_1_ degradation after 44 h of incubation in an in vitro study [157]. Similarly, another study reported that *Nocardia corynebacteroides* degraded AFB_1_ [158], decreased lesion scores in the duodenum of chickens, reduced residual AFB_1_ in the liver, and improved growth performance compared to chickens fed an AFB_1_-contaminated diet without *Nocardia corynebacteroides* [159]. However, the specific less toxic metabolite produced by *Nocardia corynebacteroides* has not been investigated, and the degradation rate of AFB_1_ by *Nocardia corynebacteroides* is influenced by heat and proteinase, which affect enzyme efficiency [158].

### 4.3. Multi-Component Mycotoxin-Detoxifying Agent

To maximize the mitigation of the negative impacts of AFB_1_ on the intestinal health and growth performance of monogastric animals, multi-component mycotoxin-detoxifying agents can be used in chicken and pig feeds (Table 7) [57,96,160]. Naturally contaminated feedstuffs often contain multiple mycotoxins; a global survey of over 70,000 samples found that 64% of sampled feeds and feedstuffs were contaminated with more than one mycotoxin [1]. Multi-component mycotoxin-detoxifying agents may be more effective in mitigating the impacts of AFB_1_ and other mycotoxins than single-component mycotoxin-detoxifying agents, by combining adsorption and detoxification properties and positive effects on intestinal health, offering broader coverage when mycotoxin contamination is unspecified.

The specificity of mycotoxin-adsorbing agents further supports the use of multi-component mycotoxin-detoxifying agents. Clay compounds, common and effective mycotoxin-adsorbing agents for AFB_1_, have a low affinity for mycotoxins such as DON; therefore, the use of clay in combination with additional mycotoxin-detoxifying agents could be advantageous for use in situations of AFB_1_ co-contaminated with other mycotoxins [42]. The addition of β-glucans from yeast and algae may be more effective than minerals and clay to mitigate the impacts of mycotoxins other than AFB_1_ [161]. A previous in vitro study reported that yeast cell walls had a higher affinity for adsorption to DON when compared to bentonite, cellulose, and activated charcoal, exemplifying that no single mitigation product will be appropriate in every situation [132]. Additionally, the combined use of mycotoxin-degrading enzyme supplementation with bacteria including adsorbing agents (*Lactobacillus casei* and *Enterococcus faecalis)* and biotransforming agents (*Bacillus subtilis* and *Candida utilis*) showed beneficial effects on intestinal health and growth, thereby mitigating the negative impacts of AFB_1_ on animals [42]. Overall, multi-component mycotoxin-detoxifying agents may provide enhanced detoxification efficiency in managing AFB_1_ co-contamination with other mycotoxins.

### 4.4. Other Feed Additives That Mitigate Mycotoxin Impacts

Select non-nutritive feed additives, such as antioxidants, have been shown to help chickens and pigs mitigate the impacts of mycotoxins on growth performance by improving intestinal barrier functions and anti-oxidative capacity (Table 8) [72,129,130].

Phytobiotics offer an additional solution to counteract the adverse impacts of AFB_1_ on monogastric animals by promoting intestinal health and growth performance through their anti-inflammatory, antimicrobial, and anti-oxidative properties [72,83,106]. The phenolic compounds in phytobiotics mitigate oxidative damage to epithelial cells, support tissue repair, and enhance mucus secretion, improving overall intestinal health [74]. Tannic acid, a polyphenol known for its ability to bind polar organic compounds and prevent lipid oxidation, enhances enzyme activity and reduces oxidative stress in the liver [129]. Similarly, grape seed extract, which is rich in proanthocyanidins with strong antioxidant and anti-inflammatory effects, counters the adverse impacts of AFB_1_ by removing ROS, inhibiting lipid peroxidation in epithelial cells, and reducing pro-inflammatory cytokine production and expression in the small intestine [162,163,164].

Selenium, curcumin, and lycopene mitigate oxidative stress caused by AFB_1_ by protecting epithelial cells in the intestine and liver through distinct mechanisms [54,165,166]. Selenium, a critical component of selenoprotein enzymes such as glutathione peroxidase, supports antioxidant defenses by modulating the cellular dysfunction–apoptosis switch, decreasing pro-apoptotic protein concentrations, and increasing anti-apoptotic proteins in the liver [130,167]. Curcumin, a phenolic compound derived from the rhizome of *Curcuma longa*, exhibits powerful radical scavenging abilities due to the number and position of its hydroxyl groups [168]. Curcumin counteracts AFB_1_-induced oxidative stress by reducing protein carbonylation, lipid peroxidation, and mitochondrial permeability transition and activating antioxidant-related genes such as CAT, SOD, and glutathione S-transferase, and detoxification genes. Additionally, curcumin inhibits pyroptosis signaling in the liver [169], which may prevent intestinal cell membrane damage [54]. A previous study demonstrated that combining curcumin and a cellulosic polymer was more effective than using either alone, improving immune and intestinal parameters in chickens fed AFB_1_-contaminated diets [54]. This could be attributed to the immunomodulatory properties of curcumin [170] and the adsorptive properties of the cellulosic polymer [171] working synergistically. Lycopene, a carotenoid, reduces oxidative mitochondrial damage through the activation of NrF2 signaling pathways, stimulation of mitochondrial antioxidant capacity, and maintenance of mitochondrial biogenesis [172]. Lycopene also decreases inflammatory cytokines such as IFN-γ and IL-1β and reduces lipid oxidation by increasing the antioxidant enzyme activities and their mRNA expression in the broiler jejunum [173]. Together, these compounds could play a significant role in mitigating the impacts of AFB_1_ exposure on intestinal health, primarily by enhancing antioxidant activity and maintaining cell cycle function.

## 5. Conclusions

The GIT is the primary biological system exposed to aflatoxins upon ingestion, where their detrimental impacts first manifest in monogastric animals, including chickens and pigs. Aflatoxins, particularly AFB_1_, disrupt the intestinal microbiota, induce immune responses and the production of oxidative damage products, thereby negatively affecting intestinal morphology and reducing nutrient digestion. After AFB_1_ absorption in the small intestine of the animals, the AFB_1_ causes systemic negative impacts on animals, especially in the liver. These combined impacts of AFB_1_ reduce growth performance in animals. Based on the meta-analysis, for every 1 µg/kg·d of AFB_1_ intake per BW, average daily gain decreases by 0.13% in chickens and by 0.74% in pigs, indicating that increasing AFB_1_ intake linearly reduces animal growth.

Mycotoxin-adsorbing agents, such as clay and yeast cell walls, effectively bind AFB_1_ in the digesta, reducing its adverse impacts on intestinal health and growth performance. Additionally, biotransforming agents further support intestinal integrity, functionality, and growth by degrading AFB_1_ into less toxic metabolites in the digesta. Multi-component detoxifying agents targeting AFB_1_ could offer enhanced efficacy by adsorption and degradation together in the digesta of animals, which prevent negative impacts on intestinal health and growth performance. Naturally contaminated feedstuffs are often co-contaminated with AFB_1_ and other mycotoxins. Therefore, multi-component mycotoxin-detoxifying agents offer different binding and degrading efficiencies for mycotoxins, that could mitigate the negative impacts on intestinal health and growth performance in animals, in the context of feeds with naturally occurring AFB_1_, co-contaminated with other mycotoxins.

## Figures and Tables

**Table 2:** Intestinal morphology and nutrient digestion in chickens and pigs fed diets contaminated with aflatoxin B1 (AFB_1_). Changes were indicated using ↑ (increase) and ↓ (decrease).

Age or IBW ^1^	Experimental Period (d)	AFB_1_ (µg/kg)	Result ^2,3^	Reference
Intestinal morphology				
Chicken (d)				
1	7 to 21	600	↓ villus height, ↓ villus width, ↓ VH:CD, and ↑ crypt depth (small intestine)	[46]
1	7 to 21	600	↓ villus height, ↓ VH:CD, ↑ crypt depth, and ↑ G_2_/M cell cycle arrest (jejunum)	[59]
1	7 to 21	600	↓ villus height, ↓ VH:CD, ↓ number of absorptive cells, ↑ number of TUNEL-positive cells, and ↑ apoptotic rate in cells (jejunum)	[51]
1	20	1500	↑ lactulose/rhamnose ratio (jejunum)	[71]
1	21	60 to 120	↑ crypt depth at 60 µg/kg of AFB_1_ (jejunum) and ↓ villus height at 120 µg/kg of AFB_1_ (ileum)	[72]
1	28	0.5 mL of 20 µg/d	↑ epithelial thickness, ↑ enterocyte proliferation, ↑ epithelial plasma cell infiltration, and ↑ goblet cell proliferation (small intestine)	[73]
1	42	42	↓ villus height, ↓ VH:CD, and ↑ crypt depth (jejunum)	[42]
1	35	1000	↓ villus height, ↓villus width, ↓ VH:CD, and ↑ crypt depth (jejunum)	[74]
7	14	394 to 1574	↓ villus height, ↓ VH:CD, ↑ crypt depth, ↑ goblet cell count, and ↑ lamina propria lymphoid follicles diameter (jejunum)	[22]
11	19	250	↓ villus height, ↓ villus width, ↓ VH:CD, and ↑ crypt depth (jejunum)	[27]
Pig (kg)				
6	48	180	↓ villus height (jejunum)	[43]
7	30	500	↓ fold size and ↑ villus height (small intestine)	[55]
Not available ^4^	48 h	40	↓ rate of intestinal cell viability, ↑ the % of necrotic cell, ↑ late apoptotic cell, and ↑ early apoptotic cell (jejunal cell culture)	[25]
Not available ^4^	12 h	30	↓ fluorescent intensity of Bcl-2 and ↑ fluorescent intensity of the ratio of BaX to Bcl-2 (jejunal cell culture)	[24]
		40 to 60	↓ cell viability (jejunal cell culture)	
Nutrient digestion				
Chicken (d)				
1	20	1500	↓ AID of GE, CP, Asp, Thr, Pro, Gly, Ala, Cys, Val, Ile, Leu, Tyr, Phe, and His	[71]
1	21	70 to 750	↑ ATTD of EE at 750 µg/kg of AFB_1_	[17]
1	273	2500 to 3910	↓ Retention of DM, CP, and EE	[67]
11	19	250	↓ ATTD of GE, CP, and EE	[27]
Pig (kg)				
7	31	180	↓ AID of CP	[45]
38	102	102	↓ ATTD of DM, GE, and EE	[26]

^1^ IBW = initial body weight. ^2^ Result description based on the comparison between diets contaminated with aflatoxins and control diet. ^3^ VH:CD = villus height-to-crypt depth ratio; AID = apparent ileal digestibility; ATTD = apparent total tract digestibility; DM = dry matter; GE = gross energy; CP = crude protein; EE = ether extract; Bcl-2 = B-cell lymphoma protein 2; Bax = Bcl-2-associated X protein. ^4^ In vitro, porcine jejunal epithelial cells were used.

**Table 7:** Growth performance of chickens and pigs fed diets contaminated with aflatoxin B1 (AFB_1_) including a multi-component mycotoxin-detoxifying agent.

Age or IBW ^1^	Experimental Period (d)	AFB_1_ (µg/kg)	Type	Level (%)	Growth Performance ^2^ (% Change)						Reference
					ADG		ADFI		G:F		
					vs. AFB_1_^3^	vs. Control ^4^	vs. AFB_1_	vs. Control	vs. AFB_1_	vs. Control	
Chicken (d)											
1	42	200	Clay (bentonite) + yeast cell wall	0.20	0.17	−3.09	0.90	−0.57	−0.72	−2.53	[57]
1	35	1000	Clay (bentonite and HSCA) + oligomannose	0.05	7.77 **	−8.37 **	4.65	0.10	2.97	−8.46 **	[74]
1	42	42	Adsorbing agent ^5^ [Clay (montmorillonite), *Lactobacillus casei*, and *Enterococcus faecalis*)] + biotransforming agent ^6^ (*Bacillus subtilis, Candida utilis*, and mycotoxin-degrading enzyme)	0.50	5.35	−8.47	−8.47	−3.92	15.10	1.26	[42]
				0.10	8.42	−12.65	24.44 **	−1.12	24.44 **	9.19	
				0.15	9.75 **	−11.16	24.44 **	0.10	24.44 **	8.69	
1	42	600	Adsorbing agent [clay (bentonite), activated charcoal, *Lactobacillus* sp., and *Bifidobacterium* sp.] + biotransforming agent (*Bacillus* sp.)	0.10	18.75 **	−5.00 **	8.14 **	−2.11	9.81 **	−2.96 **	[48]
1	37	40	Adsorbing gent (*Lactobacillus acidophilus*) + biotransforming agent (*Bacillus subtitlis*)	0.005	3.45 **	7.14 **	−5.68 **	−1.19 **	9.68 **	8.43 **	[105]
1	42	500	Adsorbing gent (*Streptococcus salivarius sp. Thermophilus*, *Lactobacillus* spp. ^7^, *Bifidobacterium bifidum*, *Enterococcus faecium*, and *Candida pintolopesii*) + biotransforming agent (*Aspergillus oryzae*)	0.50	5.66 **	−6.67 **	−2.97 **	−4.85 **	8.89 **	−1.90 **	[106]
Pig (kg)											
7	30	500	Yeast cell wall + calcium carbonate	0.10	15.15 **	−7.32	−3.77	−22.73	19.67	19.94	[55]
9	42	150	Clay (sodium bentonite and sepiolite) + dried yeast	0.011	4.10	−6.86	0.53	−6.50	4.32	−0.39	[124]
			Clay (sodium bentonite and sepiolite) + yeast culture	0.015	2.12	−8.98	0.66	−6.37	1.80	−2.79	
6	32	217	Clay (bentonite) + yeast cell wall	0.20	6.76	−11.76	6.76	−11.76	−1.04	1.33	[66]
				0.40	19.93 *	−0.88	19.93 *	−0.88	1.86	4.30	

^1^ IBW = initial body weight. ^2^ Asterisk marks (*, **) represent statistical tendency (*p* < 0.10) and significant difference (*p* < 0.05), respectively. ^3^ The percentage increase or decrease in the average daily gain (ADG), average daily feed intake (ADFI), and gain-to-feed ratio (G:F) was determined in aflatoxin contamination with mitigation agents relative to the aflatoxin contamination group. ^4^ The percentage increase or decrease in the ADG, ADFI, and G:F was determined in aflatoxin contamination with mitigation agents relative to the control group. ^5^ Adsorbing agent = *Lactobacillus casei* (1.0 × 10^8^ CFU/g) and *Enterococcus faecalis* (1.0 × 10^10^ CFU/g) were included. ^6^ Biotransforming agent *= Bacillus subtilis* (1.0 × 10^8^ CFU/g) and *Candida utilis* (1.0 × 10^8^ CFU/g) were included. ^7^ *Lactobacillus* spp. included *Lactobacillus delbrueckii* subsp. *bulgaricus*, *Lactobacillus acidophilus*, *Lactobacillus plantarum*, and *Lactobacillus rhamnosus*.

**Table 8:** Growth performance of chickens and pigs fed diets contaminated with aflatoxin B1 (AFB_1_) including other feed additives that mitigate mycotoxin impacts.

Age or IBW ^1^	Experimental Period (d)	AFB_1_ (µg/kg)	Type	Level (%)	Growth Performance ^2^ (% Change)						Reference
					ADG		ADFI		G:F		
					vs. AFB_1_^3^	vs. Control ^4^	vs. AFB_1_	vs. Control	vs. AFB_1_	vs. Control	
Chicken (d)											
1	35	1000	Clay (bentonite and HSCA) + oligomannose + phytobiotics	0.05	7.88 **	−8.27 **	2.74	−1.73	5.01 **	−6.65 **	[74]
1	42	100	Clay (sodium bentonite) + gention violet	0.50	11.60 **	−27.99 **	3.50 **	−24.10 **	7.83 **	−5.14 **	[96]
				1.00	10.61 **	−28.63 **	4.91 **	−23.06 **	5.44 **	−7.24 **	
			Clay (sodium bentonite) + acetic acid	0.50	13.02 **	−27.08 **	3.65 **	−23.99 **	9.05 **	−4.07 **	
				1.00	8.14 **	−30.23 **	−2.12 **	−28.22 **	10.48 **	−2.81 **	
1	35	38	Nano-composite magnetic graphene oxide + chitosan	0.25	6.56 **	−2.36	7.86 **	5.24	−1.20	−0.28	[109]
				0.50	8.44 **	−0.65	2.11	−0.36	6.19 **	−0.28	
1	35	1000	Phytobiotics	0.05	7.13 **	−8.90 **	1.52	−2.89	5.53 **	−6.19 **	[74]
1	44	122	Sporoderm-broken spores of *Ganoderma lucidum*	0.02	6.55 **	3.54 **	4.41 **	0.92	2.05 *	1.82 **	[88]
1	42	600	Milk thistle (*Silybum marianum*)	1.00	12.50 **	−10.00 **	10.47 **	0.00	1.84 **	−10.00 **	[48]
1	28	1000	Grapeseed extract	0.025	19.43 **	−21.57 **	12.50 **	2.65 **	6.67 **	−2.47 **	[83]
				0.050	18.44 **	−22.22 **	16.07 **	3.54 **	3.38	−5.47 **	
1	21	60	Tannic acid	0.025	2.63 **	5.41 **	6.25 **	8.51 **	1.98 **	−7.77 **	[72]
				0.050	2.63 **	5.41 **	4.17 **	6.38 **	3.64 **	−6.26 **	
1	21	100	Lycopene	0.020	6.52 **	−3.92 **	3.45 **	−2.17 **	2.97 **	−1.79 **	[100]
1	42	500	Licorice extract	0.30	7.55 **	−5.00 **	0.00	−1.94 **	7.55 **	−3.12 **	[106]
				0.60	5.66 **	−6.67 **	−0.99 **	−2.91 **	6.72 **	−3.87 **	
			Poultry litter biochar	0.50	3.77 **	−8.33 **	0.99 **	−0.97 **	2.76 **	−7.43 **	
1	42	50	Calcium propionate	0.25	4.12	−0.09	9.87	−0.76	−5.24	0.67	[103]
				0.50	4.51	0.28	10.40	−0.28	−5.33	0.57	
		100	Calcium propionate	0.25	5.79	−0.28	15.67	−1.42	−8.54	1.15	
				0.50	6.29	0.19	16.89	−0.38	−9.07	0.57	
1	21	2000	Curcumin	0.20	19.80 **	−10.21 **	1.33	4.83 **	−0.35	−19.77 **	[54]
1	21	2000	Cellulosic polymer + curcumin	0.50	23.22 **	−7.64 **	−2.12 **	1.27	24.20 **	0.00	[54]
1	42	600	Algae (*Spirulina platensis*)	1.00	12.50 **	−10.00 **	3.49 **	−6.32 **	8.71 **	−3.93 **	[48]
Pig (kg)											
7	40	500	Vegetable biocholine	0.08	6.55	−9.62	0.56	−11.11 **	5.95	1.68 **	[112]
9	30	320	Grape seed waste	8.00	51.05 **	−11.84	-	-	-	-	[56]
11	28	420	Selenium	0.006	2.17	−9.62	5.26	−11.50	−2.93	2.13	[114]
		840	Selenium	0.006	10.71 **	−40.38	1.49	−39.82	9.09	−0.93	
		840	Folic acid	0.020	32.14 **	−28.85	23.88	−26.55	6.67	−3.13	

^1^ IBW = initial body weight. ^2^ Asterisk marks (*, **) represent statistical tendency (*p* < 0.10) and significant difference (*p* < 0.05), respectively. ^3^ The percentage increase or decrease in the average daily gain (ADG), average daily feed intake (ADFI), and gain-to-feed ratio (G:F) was determined in aflatoxin contamination with mitigation agents relative to the aflatoxin contamination group. ^4^ The percentage increase or decrease in the ADG, ADFI, and G:F was determined in aflatoxin contamination with mitigation agents relative to the control group.

## Data Availability

The original contributions presented in this study are included in the article. Further inquiries can be directed to the corresponding author(s).
